# Mapping the natural course of comorbid bipolar disorder via integration of multimodal data (MapCoBD): a study protocol

**DOI:** 10.3389/fnhum.2026.1924416

**Published:** 2026-10-14

**Authors:** Stefano Comai, Maria Morena, Luca Steardo, Alessio Squassina, Claudia Pisanu, Anna Meloni, Donatella Congiu, Silvia Lazzardi, Marco Pinna, Martina Contu, Sofia Nasini, Benedetta Barzon, Angelica Maria Zuliani, Stefano Dall'Acqua, Antonella Balestrieri, Patrizia Campolongo, Ilaria Pullano, Anna Maria Iazzolino, Renato de Filippis, Federica Pinna, Luca Saba, Mirko Manchia

**Affiliations:** 1 Department of Pharmaceutical and Pharmacological Sciences, University of Padova, Padova, Italy; 2 Department of Psychiatry, McGill University, Montreal, QC, Canada; 3 Department of Physiology and Pharmacology, Sapienza University of Rome, Rome, Italy; 4 Neuropharmacology Unit, IRCCS Fondazione Santa Lucia, Rome, Italy; 5 Department of Mental Health, Azienda Sanitaria Locale Salerno, Salerno, Italy; 6 Section of Neuroscience and Clinical Pharmacology, Department of Biomedical Sciences, University of Cagliari, Cagliari, Italy; 7 Section of Psychiatry, Department of Medical Sciences and Public Health, University of Cagliari, Cagliari, Italy; 8 Unit of Clinical Psychiatry, Department of Medical Specialties, University Hospital Agency of Cagliari, Cagliari, Italy; 9 Unit of Radiology, Department of Health Services, University Hospital Agency of Cagliari, Cagliari, Italy; 10 Section of Radiology, Department of Medical Sciences and Public Health, University of Cagliari, Cagliari, Italy; 11 Psychiatry Unit, Department of Health Sciences, University Magna Graecia of Catanzaro, Catanzaro, Italy; 12 University Hospital “Renato Dulbecco” of Catanzaro, Catanzaro, Italy; 13 Department of Pharmacology, Dalhousie University, Halifax, NS, Canada

**Keywords:** biomarkers, endocannabinoids, inflammation, microRNA, neuroimaging, attention deficit hyperactivity disorder, borderline personality disorder

## Abstract

**Background:**

Bipolar disorder (BD) is a chronic, disabling psychiatric condition with a high global disease burden. Its course is marked by alternating episodes of mania and depression, with varying degrees of severity. While cyclicity is a defining feature, clinical patterns show significant inter-individual and intra-individual variability. Although naturalistic studies have described the course of BD, the impact of psychiatric comorbidities, particularly attention deficit hyperactivity disorder (ADHD) and borderline personality disorder (BPD), remains underexplored. MapCoBD is a 12-month, naturalistic observational study whose primary objective is to compare the longitudinal clinical course of BD among individuals with classic episodic BD, BD with comorbid ADHD, and BD with comorbid BPD. Secondary objectives are to characterize the clinical, neuropsychological, neuroimaging, and molecular features associated with these distinct clinical courses.

**Content:**

This project will recruit 150 BD individuals divided into three groups: (1) classic episodic BD (epiBD, *n* = 50), (2) BD with comorbid ADHD (BD-ADHD, *n* = 50), and (3) BD with comorbid BPD (BD-BPD, *n* = 50). Assessments will be conducted at baseline (T0), 6 months (T1), and 12 months (T2), including: mood symptom ratings, neuropsychological testing, fasting blood sampling (for serum, plasma, and microRNA), and 3T structural brain MRI scans. Daily mood ratings will also be collected prospectively throughout the study. The primary longitudinal clinical endpoint will be illness burden, defined as the proportion of evaluable follow-up days spent with moderate-to-severe psychopathological symptoms, based on prospective NIMH Life Chart Method assessments. Daily affective ratings will additionally be used to characterize mood instability. At each time point, peripheral biomarkers implicated in BD pathophysiology, including endocannabinoid (eCB) signaling, inflammatory cascade (IC), and epigenetic regulation (microRNA), will be quantified. Clinical, neuroimaging, and molecular data will also be integrated using supervised machine-learning approaches to explore the classification of clinical group status and the prediction of longitudinal mood instability and illness burden.

**Conclusions:**

This study will determine whether ADHD and BPD comorbidity is associated with distinct longitudinal patterns of illness burden and mood instability in BD. The integration of clinical, neuroimaging, and molecular data may identify group-specific multimodal signatures and candidate predictors of clinical course, which will require independent validation before clinical application.

## Introduction

Bipolar disorder (BD) is a serious, chronic non-communicable psychiatric illness ranking in the top 10 causes of disability and mortality worldwide (Ferrari et al., 2016; Lai et al., 2024). BD typically begins in adolescence or early adulthood and follows a life-long course characterized by alternating episodes of mania or hypomania and depression, often accompanied by psychotic symptoms, cognitive impairment, functional decline, and medical and psychiatric comorbidities (McIntyre et al., 2020).

The longitudinal course of BD is characterized by mood instability and recurrent shifts in affective states (McIntyre et al., 2022). Attempts to describe the natural fluctuations of mood in episodic BD (epiBD) using sophisticated methods for episode forecasting, including approximate entropy and machine learning approaches, have shown that the onset of a new episode is associated with greater irregularity in mood and that evening energy plays an important deterministic role (Ortiz et al., 2018). However, there is consensus that epiBD is characterized by a later age of onset, by low rates of comorbid conditions and by familial loading for BD (Suppes et al., 2000; Alda, 2004).

Cyclicity is a key component of the clinical course of BD, but its patterns vary substantially both between individuals (inter-individual heterogeneity) and longitudinally over time within the same individual (intra-individual heterogeneity; Tondo et al., 2014). Some, but not all, determinants of this heterogeneity have been described, including duration and severity of depression, stability of depression, duration and severity of hypomania, stability of mania, mixed symptoms, and mania-to-depression or depression-to-mania phase switching (Oliva et al., 2025). However, the biological determinants of these distinct longitudinal trajectories remain poorly understood, particularly with respect to neuroimaging and peripheral biomarkers (Smeland et al., 2025). Furthermore, and importantly, although naturalistic studies have described diverse forms of clinical course in typical BD, the impact of psychiatric comorbidities on the longitudinal evolution of BD has rarely been systematically investigated. Attention deficit hyperactivity disorder (ADHD) and borderline personality disorder (BPD) are relatively frequent comorbidities and are associated with an unfavorable illness course in BD (Frías et al., 2016; Schiweck et al., 2021). Specifically, individuals with BD and comorbid BPD are characterized by greater psychiatric complexity, elevated suicide-related morbidity, and increased longer-term hospital utilization compared to typical BD (Viana Neto et al., 2026). Similarly, people living with BD and comorbid ADHD show a severe and burdensome clinical picture, with a lower quality of life, a higher number of mood episodes, an increased prevalence of substance abuse and dependence, and worse overall functioning (Salvi et al., 2021).

Clinical characterization alone, however, may not fully explain why individuals with apparently similar BD diagnoses follow markedly different longitudinal trajectories. Overlapping manifestations such as mood instability, impulsivity, attentional difficulties, and stress sensitivity may reflect partly shared or distinct underlying biological processes across epiBD, BD-ADHD, and BD-BPD. Investigating biological mechanisms alongside prospective clinical course is therefore important to determine whether these comorbid profiles represent only differences in symptom severity or are associated with distinguishable neurobiological features. Repeated biological assessments may also help differentiate relatively stable comorbidity-related features from state-dependent changes accompanying fluctuations in mood and illness burden.

epiBD has traditionally provided the main reference for characterizing the cyclicity and longitudinal course of the disorder (Koukopoulos et al., 2013), whereas considerably less is known about BD trajectories in the presence of ADHD or BPD. Evidence concerning the neuroimaging and biological correlates of these comorbid presentations is also sparse and predominantly cross-sectional. Indeed, only one study has shown that individuals with BD and comorbid ADHD exhibit significant hyperactivation of the anterior cingulate cortex compared with individuals with only BD (Hegarty et al., 2012). Consequently, it remains unclear whether the biological alterations associated with these comorbidities represent relatively stable features, fluctuate with mood state, or relate to subsequent illness burden. This gap supports the need for multimodal longitudinal protocols capable of capturing both the clinical and biological architecture of BD heterogeneity. In this context, we propose a protocol for investigating three key biological systems that may be particularly relevant to mood regulation, stress responsivity, and neurobiological heterogeneity in BD and its comorbidities: the endocannabinoid (eCB) system, inflammation as assessed through the levels of inflammatory cytokines, and the tryptophan (Trp)-kynurenine (Kyn) pathway (KP). The eCB system has emerged as a critical modulator of synaptic plasticity and emotional processing (Morena et al., 2016), with growing evidence suggesting its dysregulation in BD (Arjmand et al., 2019). Interestingly, gene expression levels of the cannabinoid receptor gene CNR1, which encodes the cannabinoid type 1 (CB1) receptor, were reported to be higher and less variable in peripheral blood mononuclear cells of individuals with bipolar disorder type I (BD I) during mania than during depression (Escelsior et al., 2022). Candidate gene studies have explored the role of genetic variants within components of the eCB system although findings have been conflicting and generally not replicated (Minocci et al., 2011; Monteleone et al., 2010; Pisanu et al., 2013). However, pathway analysis of the large-scale genome-wide association study (GWAS) of the Psychiatric Genomics Consortium Bipolar Disorder (PGC-BD) showed enrichment for gene sets of the eCB signaling (Stahl et al., 2019), supporting the hypothesis that this system may contribute to BD vulnerability and clinical expression.

The inflammatory hypothesis of BD has gained substantial empirical support, with meta-analyses consistently demonstrating elevated pro-inflammatory cytokines (including IL-6 and TNF-α) across mood states, particularly during acute episodes (Munkholm et al., 2013). These inflammatory markers have been linked not only to symptom severity but also to cognitive dysfunction and treatment resistance. Notably, both ADHD and BPD have been independently associated with immune system dysregulation (Dunn et al., 2019; MacDowell et al., 2020; Saccaro et al., 2021), indicating that inflammatory biomarkers may help identify biologically distinct BD subgroups and improve prediction of longitudinal course. The KP of Trp metabolism represents another promising biomarker candidate, serving as a critical interface between immune activation and neuroplasticity (Comai et al., 2020; Pocivavsek et al., 2024). Under conditions of inflammation or stress, indoleamine 2,3-dioxygenase (IDO) and/or tryptophan 2,3-dioxygenase (TDO) activation shifts Trp metabolism toward the production of neuroactive Kyn metabolites, including the neuroprotective kynurenic acid (KYNA) and the neurotoxic quinolinic acid (QA; Comai et al., 2020; Pocivavsek et al., 2024; Savitz et al., 2015). Alterations in the KP have been implicated in BD pathophysiology as well as in treatment response. Emerging evidence that the KP may also be dysregulated in ADHD (Cavaleri et al., 2024) and BPD (Javelle et al., 2025) may suggest that BD comorbid with ADHD and/or BPD may be associated with a more complex or more severe biological disturbance involving immune-metabolic pathways. Finally, although the evidence is still limited, the eCB system and KP are likely interconnected through shared inflammatory and stress-response mechanisms. During inflammation, cytokines activate IDO, diverting Trp toward Kyn and reducing serotonin (5-HT) synthesis, while simultaneously stimulating eCB signaling [e.g., 2-arachidonoylglycerol (2-AG) and anandamide (AEA)] as a compensatory response. In depression, dysregulation of the eCB system likely at the level of catabolic enzymes (e.g., FAAH, MAGL) and of Kyn metabolites suggests a coordinated but potentially maladaptive response to inflammation and/or stress (Comai et al., 2024). BD involves multiple and partly overlapping biological mechanisms, including alterations in neurotransmission, circadian regulation, neurotrophic and synaptic plasticity, mitochondrial function, oxidative stress, immune signaling, and epigenetic regulation (McIntyre et al., 2020; Smeland et al., 2025). Within this broader biological landscape, MapCoBD prioritizes inflammatory signaling, the endocannabinoid system, the tryptophan–kynurenine pathway, and microRNA profiles because they provide complementary and potentially interconnected measures of immune activation, stress responsivity, neuroactive metabolism, and gene regulation. Several of these processes have also been implicated in ADHD and BPD, making them particularly relevant to the planned comparison of the three clinical groups (Dunn et al., 2019; MacDowell et al., 2020; Saccaro et al., 2021; Comai et al., 2020; Pocivavsek et al., 2024; Savitz et al., 2015). Moreover, these measures can be repeatedly assessed in peripheral blood and integrated with longitudinal clinical and neuroimaging data. Thus, the selected panel is not intended to provide an exhaustive account of BD biology, but rather a focused and feasible framework to examine whether coordinated alterations across these systems distinguish comorbid clinical profiles or track mood instability and illness burden over time. Taken together, these findings suggest that ADHD and BPD are associated with a more complex clinical course of BD and that the joint investigation of neuroimaging and biological pathways may provide a more integrated understanding of the mechanisms linking mood instability, stress sensitivity, and psychiatric comorbidity. However, available evidence largely derives from separate, cross-sectional studies focused on a single comorbidity or biological domain. To our knowledge, no longitudinal study has directly compared epiBD, BD-ADHD, and BD-BPD while repeatedly assessing clinical course together with neuroimaging and molecular measures. It therefore remains unclear whether the reported biological alterations represent stable features associated with comorbidity, vary with mood state, or are related to subsequent illness burden. MapCoBD was designed to address this unmet need through prospective clinical monitoring and repeated multimodal assessments.

Accordingly, the primary research question of MapCoBD is whether the 12-month clinical course of BD, operationalized primarily as time spent ill with moderate-to-severe psychopathological symptoms, differs among individuals with classic episodic BD, BD with comorbid ADHD, and BD with comorbid BPD. Secondary research questions are whether these groups differ cross-sectionally and longitudinally in clinical and neuropsychological characteristics, structural and functional neuroimaging measures, microRNA expression, inflammatory and endocannabinoid markers, and tryptophan–kynurenine pathway measures. The study will also explore whether combinations of these clinical and biological features can classify the three clinical groups and predict subsequent mood instability and illness burden. We hypothesize that participants with BD-ADHD or BD-BPD will show a greater longitudinal illness burden and mood instability than participants with epiBD. We further expect BD-BPD to be characterized by greater affective instability and impulsivity, whereas BD-ADHD is expected to show more prominent attentional and neuropsychological impairment. Potential group-specific neuroimaging and molecular profiles will also be examined through exploratory, hypothesis-generating analyses. Given the limited and partly inconsistent evidence, no predefined direction of effect is proposed for individual microRNA, endocannabinoid, inflammatory, kynurenine-pathway, or neuroimaging measures.

## Methods

### Study objectives and hypotheses

MapCoBD is a 12-month naturalistic observational study including 150 individuals with BD divided into three clinically defined groups: classic episodic BD (epiBD; *n* = 50), BD with comorbid ADHD (BD-ADHD; *n* = 50), and BD with comorbid BPD (BD-BPD; *n* = 50). The primary objective is to compare the longitudinal clinical course of BD across the three groups. The primary longitudinal clinical endpoint is illness burden, defined as the proportion of evaluable follow-up days spent with moderate-to-severe psychopathological symptoms, based on prospective NIMH Life Chart Method assessments. Mood instability, derived from daily affective ratings, will be evaluated as a secondary clinical outcome. The secondary objectives are to identify cross-sectional and longitudinal differences among the three groups in: (1) clinical and neuropsychological characteristics; (2) structural and functional neuroimaging measures; (3) microRNA expression profiles; and (4) peripheral markers related to inflammatory signaling, the endocannabinoid system, and the tryptophan–kynurenine pathway. These objectives will be examined through three prespecified comparisons: epiBD vs. BD-ADHD, epiBD vs. BD-BPD, and BD-ADHD vs. BD-BPD. Exploratory objectives are to integrate clinical, neuroimaging, and molecular data to identify group-specific multimodal signatures, classify participants according to clinical group, and evaluate whether multimodal features predict subsequent mood instability or illness burden. The primary clinical hypothesis is that both comorbid groups will show a greater longitudinal illness burden and mood instability than the epiBD reference group. We further hypothesize that BD-BPD will be associated with greater affective instability and impulsivity, whereas BD-ADHD will be associated with more prominent attentional and neuropsychological impairment. Analyses of individual biological and neuroimaging measures will test for group and group-by-time differences without assuming a predefined direction of effect. An overview of the MapCoBD study design, assessment schedule, and analytical framework is provided in Figure 1.

**Figure 1:**
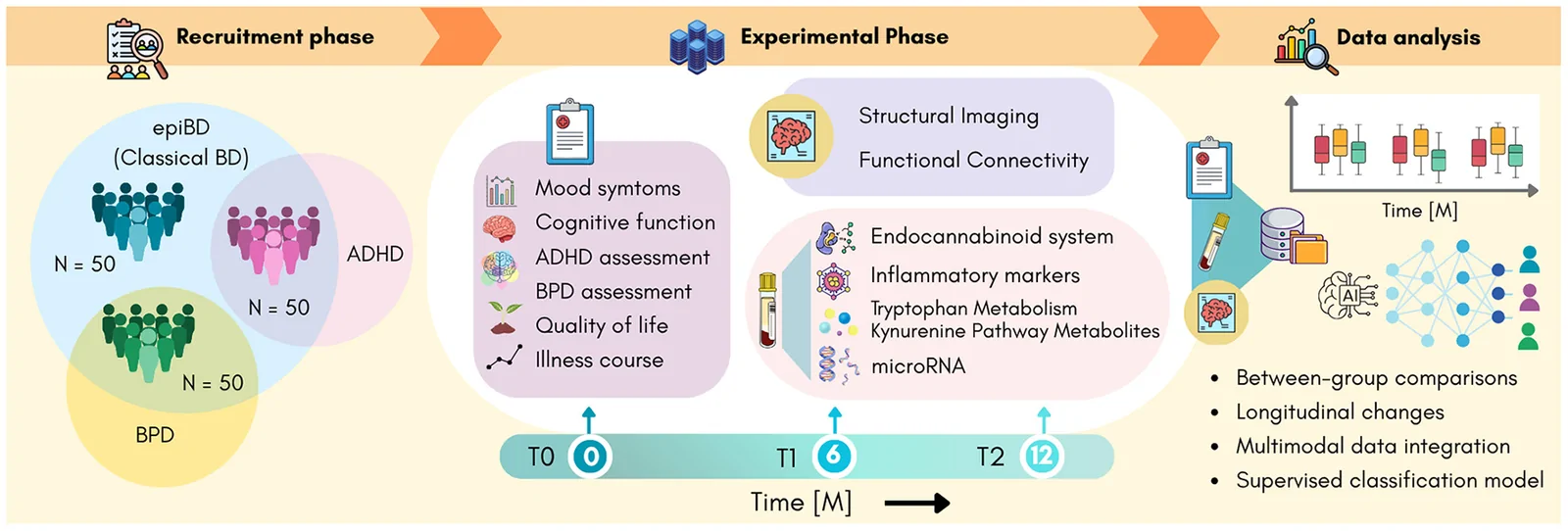
Overview of the MapCoBD study design. A total of 150 participants with BD will be recruited into three clinically defined groups: classic episodic BD (epiBD; *n* = 50), BD with comorbid attention-deficit/hyperactivity disorder (BD-ADHD; *n* = 50), and BD with comorbid borderline personality disorder (BD-BPD; *n* = 50). Participants will undergo clinical and neuropsychological assessments, neuroimaging, and molecular and peripheral biomarker assessments at baseline (T0), 6 months (T1), and 12 months (T2). Multimodal data will be analyzed to assess between-group and longitudinal differences, integrating clinical, neuroimaging, and molecular features.

### Study population and clinical assessments

#### Recruitment and group allocation

The study will include a total of 150 participants diagnosed with BD recruited at two clinical sites, the University of Cagliari and the University of Catanzaro. Participants will be assigned to three equal groups of 50 individuals according to their comorbid diagnostic profiles: epiBD, BD-ADHD, and BD-BPD. All participants will be assessed at baseline (T_0_), 6 months (T_1_), and 12 months (T_2_). As this is a naturalistic observational study, pharmacological treatments will not be standardized. At each assessment, detailed information will be collected on medication class, compound, dose, treatment duration, adherence, and any treatment initiation, discontinuation, or dose modification occurring during follow-up. Concomitant psychological interventions and other potentially relevant time-varying factors, including substance use, smoking status, body mass index, sleep quality, and medical comorbidities will also be recorded.

#### Diagnostic assessment

Primary diagnosis of BD will be established according to DSM-5 criteria and confirmed using the Italian version of the Structured Clinical Interview for DSM-5—Clinician Version (SCID-5-CV; Fossati and Borroni, 2017). The previous clinical course of BD will be reconstructed using clinical interviews, review of available medical records, and the NIMH Life Chart retrospective method (Leverich and Post, 1998). Presence of ADHD or BPD will be longitudinally established as a concomitant or subsequent manifestation to the onset of BD. Comorbid ADHD diagnosis will be established using the adult ADHD Self-Report Scale (Kessler et al., 2005), the Wender Utah Rating Scale (25-item version; Ward, 1993), and the Diagnostic Interview for ADHD in adults (Ramos-Quiroga et al., 2019). Diagnosis of ADHD will be also corroborated by interviews with informants (relative/partner). Similarly, comorbid BPD diagnosis will be assessed using the SCID-5 Alternative Model for Personality Disorders Modules I and II (Bender et al., 2018; Skodol et al., 2018), the Zanarini Rating Scale for Borderline Personality Disorder (Zanarini, 2003), the Clinical Global Impression-BPD, and the Borderline Personality Disorder Severity Index (BPDSI-IV; Giesen-Bloo et al., 2010).

#### Clinical and neuropsychological assessments

At T_0_, T_1_, and T_2_, mood and anxiety symptoms will be assessed with the Italian versions of the Hamilton Depression Rating Scale (21 items; Hamilton, 1960), Hamilton Anxiety Rating Scale (21 items; Hamilton, 1959), Young Mania Rating Scale (Young et al., 1978), and Clinical Global Impression-Severity (Guy, 1976). Neuropsychological function will be evaluated using the Brief Assessment of Cognition in Affective Disorders (Keefe et al., 2004), while impulsivity and quality of life will be assessed using the Barratt Impulsiveness Scale version 11 (Fossati et al., 2002) and the Short-Form Health Survey (12 items; Ware et al., 1996), respectively. Diagnostic interviews will not be routinely repeated at T_1_ and T_2_ unless clinically indicated.

#### Prospective mood and illness-course monitoring

All participants will complete daily affective ratings throughout the 12-month study period to characterize mood instability. The clinical course will also be prospectively captured using the NIMH Life Chart Method to derive time-based indices of illness burden (Post et al., 2003). Using the prospective NIMH Life Chart Method, each evaluable day will be classified according to the presence or absence of moderate-to-severe psychopathological symptoms. Longitudinal illness burden will be calculated separately for months 0-12 as the number of days meeting this criterion divided by the total number of evaluable days within each interval. The resulting measure will range from 0, indicating no days with moderate-to-severe symptoms, to 1, indicating moderate-to-severe symptoms throughout the entire evaluable interval. Mathematically, this measure corresponds to the normalized area under the curve of the daily binary illness indicator.

#### Inclusion and exclusion criteria

The following inclusion and exclusion criteria will be applied:

Inclusion criteria:

1. Diagnosis of BD type 1 or type 2 according to the DSM-5 criteria;
2. Age between 18 and 70 years old;
3. Presence of an episodic pattern of BD (epiBD) in *N* = 50;
4. Presence of an additional diagnosis of ADHD according to the DSM-5 criteria in *N* = 50;
5. Presence of an additional diagnosis of BPD according to the DSM-5 criteria in *N* = 50.

Exclusion criteria:

1. DSM-5 criteria for psychiatric disorders other than BD, ADHD and BPD;
2. History of closed head injury resulting in loss of consciousness;
3. Untreated active medical illness (e.g., diabetes);
4. History of neurological disorders;
5. Magnetic Resonance Imaging (MRI) contraindications (metal implants, pacemakers etc.);
6. Presence of cognitive impairment (severity level of mild cognitive impairment) or intellectual disability that would interfere with study procedures;
7. Current cannabis use disorders.

### Brain imaging protocol

MRI data will be acquired at both clinical sites using 3 Tesla Philips “Achieva dStream” scanners (Philips, Best, the Netherlands; peak amplitude = 33 mT/m; slew rate = 125 mT/m/s) equipped with a 16-channel head coil. The protocol will include a diffusion-weighted imaging (DWI) sequence for DTI reconstruction, which will be acquired with the following parameters: echo time = 83.147 ms; repetition time = 3,249.39 ms; matrix = 128 × 128 × 54; resolution = 1.75 × 1.75 × 2.5 mm; diffusion directions = 34; *b*-values = 0 and 800 s/mm^2^. Functional connectivity analysis will be performed with CONN-fMRI fc toolbox in conjunction with SPM 12 (Wellcome Department of Imaging Neuroscience, London, UK; http://www.fil.ion.ucl.ac.uk/spm/). The CONN toolbox can perform seed-based correlation analysis according to the temporal low-frequency fluctuations of BOLD signals. All the structural and functional sequences will be preprocessed using CONN's default pipeline for volume-based analysis following specific steps. Two different rs-fcMR analyses will then be conducted: (a) Regions of Interest to Region of Interest (ROI-to-ROI) and (b) Seed-to-Voxel analysis. The DTI analysis will be performed using the DSI studio. Data analysis and integration will be done at University of Cagliari.

### Biomarker and molecular methods

At each study visit, blood samples will be collected between 8:00 and 10:00 a.m. following overnight fasting. Samples will include serum, plasma, and PAXgene Blood RNA tubes (Qiagen) for microRNA analysis.

### Determination of plasma endocannabinoids

Quantification of peripheral endocannabinoids (anandamide, 2-AG, OEA, and PEA), their catabolic enzymes (FAAH and MAGL), and receptor targets (CB1, CB2, PPAR-α, TRPV1) will be performed using liquid chromatography tandem mass spectrometry (LC-MS/MS), PCR and western blotting as previously described (Qi et al., 2015; Gray et al., 2015; Vecchiarelli et al., 2021).

### MicroRNA

MicroRNAs will be extracted using the RNeasy Mini Kit and processed for miRNome analysis following DNase treatment and RNA quality verification via the NanoDrop spectrophotometer. After library preparation using the Qiaseq microRNA Library Kit (Qiagen) and library quality control, sequencing will be performed with a NextSeq 2000 (Illumina) system.

### Quantification of plasma inflammatory markers

A panel of 120 inflammatory factors, growth factors, and chemokines will be measured using the Quantibody^®^ Human Cytokine Antibody Array 2000 multiplex (RayBiotech, Norcross, GA, USA) according to the manufacturer's guidelines.

### Quantification of plasma Trp, 5-HT, and Kyn

Trp, 5-HT, and Kyn will be analyzed using high-performance liquid chromatography (HPLC) with dual detection: a fluorometric detector for Trp and 5-HT (excitation: 285 nm, emission: 345 nm) and a UV–Vis detector for Kyn (absorption wavelength: 360 nm). The separation of the analytes will be performed isocratically at 0.8 mL/min using a mobile phase consisting of 10% acetonitrile and 90% 0.004 M phosphate buffer (pH 3.5) and an Apollo C18 (5 μm × 250 mm × 4.6 mm) column (Sepachrom Mega Srl, Milan, Italy; Comai et al., 2022).

### LC-MS/MS analysis of 3-HK, KYNA, QA, and MLT

3-hydroxykynurenine (3-HK), KYNA, QA, and melatonin (MLT) will be quantified using a Varian LC-MS/MS system equipped with a binary Prostar pump, a 410 autosampler, and a 320-triple quadrupole mass spectrometer with an electrospray ionization (ESI) source. The mass spectrometer will operate in multiple reaction monitoring (MRM) mode, detecting 3-HK and KYNA in positive ion mode, while QuinA is analyzed in negative ion mode. Chromatographic separation will be achieved using an Agilent Eclipse XDB C8 column (3 × 150 mm, 3.5 μm) with a gradient elution of (A) water containing 1% formic acid and (B) acetonitrile (0 min: 95% A; 5 min: 30% A; 8.3 min: 10% A; 10 min: 10% A; 11 min: 95% A; 15 min: 95% A) at a flow rate of 400 μL/min. Quantification will be performed using α-methyltryptophan as an internal standard (Bravi et al., 2025).

### Enzymatic activity and functional ratios

To assess key enzymatic activities in the KP, the following ratios will be calculated as a proxy (Sapienza et al., 2024): (1) Kyn/Trp as an indicator of IDO and TDO activity; (2) KYNA/Kyn reflecting kynurenine aminotransferase II (KAT II) activity; (3) 3-HK/Kyn representing kynurenine-3-monooxygenase (KMO) activity. Finally, the KYNA/QA ratio will be calculated to evaluate the relative modulation of N-methyl-D-aspartate receptor (NMDAR) antagonism/agonism.

### Data preparation and preprocessing

Prior to statistical and exploratory machine learning (ML) analyses, all datasets will undergo standardized quality control and preprocessing procedures to ensure data consistency, reliability, and comparability across modalities. All the variables will be inspected for completeness and internal consistency. Continuous variables will be evaluated for distributional characteristics using both graphical methods and formal normality tests. Outliers will be identified through visual inspection and standardized residuals and will be verified against source data before determining whether exclusion or transformation is appropriate. Continuous variables will be standardized or normalized when required by the statistical model or ML algorithm. Categorical variables will be harmonized through consistent coding, and derived variables (as, for example, BMI) will be generated according to predefined study definitions. Longitudinal data will be organized in a long-format structure, with one observation per participant and study visit. Molecular datasets, including inflammatory biomarkers, endocannabinoid mediators, and tryptophan–kynurenine metabolites, will undergo appropriate quality control, normalization where necessary, and batch effect evaluation. Variables exhibiting strong skewness will be transformed (e.g., logarithmic transformation) to satisfy model assumptions.

MicroRNA sequencing data will undergo quality assessment of raw sequencing reads, filtering of low-quality reads, and alignment to the reference genome using validated bioinformatics pipelines. Following read quantification, lowly expressed microRNAs will be removed according to predefined expression thresholds. Expression data will subsequently be normalized to account for differences in sequencing depth and technical variability before differential expression analyses.

Structural and functional magnetic resonance imaging data will be processed using standardized neuroimaging pipelines, including quality assessment, motion correction, spatial normalization, segmentation, registration to standard anatomical space, and smoothing where appropriate. Imaging-derived phenotypes will undergo visual and automated quality control prior to statistical analysis.

Before multimodal integration and ML analyses, all datasets will be harmonized across participants using unique study identifiers. Continuous features will be standardized to comparable scales, while highly correlated variables, near-zero variance predictors, and redundant features will be removed to reduce dimensionality and minimize multicollinearity. Batch effects arising from different acquisition sessions or laboratory procedures will be evaluated and corrected when necessary, using established harmonization approaches. For machine-learning analyses, all harmonization and batch-effect correction parameters will be estimated exclusively from the corresponding training data within the nested cross-validation pipeline and subsequently applied to the held-out data without refitting.

### Missing data

Missing data patterns will be explored descriptively to determine their extent and potential mechanisms. Variables with excessive missingness or poor data quality will be excluded from downstream analyses when appropriate. When requested for ML analyses, linear mixed-effects models (LMMs) will accommodate incomplete repeated-measures data under the MAR assumption using maximum likelihood estimation. Patterns of missing data will be explored descriptively. In the case of substantial missingness (>20%), we will perform sensitivity analyses using multiple imputation by chained equations to assess the robustness of the findings.

### Power analysis

The sample size (150) was determined to ensure adequate statistical power for the primary longitudinal analysis, which will be performed using LMM to compare illness burden among the three predefined clinical groups (epiBD, BD-ADHD, and BD-BPD) with group, time, and group × time interaction as fixed effects and participant as a random intercept. Owing to the lack of previous longitudinal studies directly comparing these three populations using the proposed multimodal design, the power calculation was based on conventional repeated-measures assumptions, assuming the detection of a medium standardized effect size (Cohen's *d*, partial η^2^, or standardized regression coefficients, as appropriate). This approach was adopted as an approximate reference for the planned LMM, given the absence of prior data to inform a model-specific power simulation accounting for the exact random-effects structure and expected missing-data pattern; this is acknowledged as a limitation of the sample size estimation. We note, however, that the final analytic model will additionally benefit from using all available longitudinal observations under the missing-at-random assumption, rather than requiring complete cases as in a standard repeated-measures design, an advantage not accounted for in the present power calculation. A medium effect size was assumed, rather than a larger one, to avoid overestimating statistical power. Assuming a two-sided significance level of α = 0.05, a statistical power of 80%, and three repeated assessments over the 12-month follow-up, a minimum sample size of approximately 120 participants was estimated. To compensate for an expected 20% loss to follow-up, the planned recruitment target was increased to 150 participants (50 individuals per group).

### Statistical analysis plan

Statistical analyses will be conducted using R, MATLAB, and/or Python, in accordance with the predefined primary, secondary, and exploratory objectives. Any deviations from the statistical analysis plan or additional analyses will be identified as *post hoc* or exploratory. Statistical significance will be set at a two-sided *p*-value < 0.05.

Continuous variables will be summarized as means and standard deviations or medians and interquartile ranges, as appropriate according to their distribution. Categorical variables will be summarized using frequencies and percentages. Distributional assumptions will be assessed using graphical methods and the Shapiro–Wilk test. Variables showing marked departures from normality will be transformed or analyzed using appropriate non-parametric methods.

Before analyzing longitudinal data, baseline demographic and clinical characteristics will be compared among the three study groups (epiBD, BD-ADHD, and BD-BPD) using one-way analysis of variance (ANOVA) or Kruskal–Wallis tests for continuous variables and χ^2^ or Fisher's exact tests for categorical variables.

### Primary longitudinal analysis

As already mentioned, the primary endpoint is the longitudinal illness burden, defined as the area under the curve (AUC) of time spent with moderate-to-severe psychopathological symptoms during the 12-month follow-up, derived from the prospective NIMH Life Chart Method. The primary endpoint is longitudinal illness burden, defined as the proportion of days spent with moderate-to-severe psychopathological symptoms. It will be calculated for the 12-month follow-up interval. The outcome will range from 0, indicating no evaluable days with moderate-to-severe symptoms, to 1, indicating moderate-to-severe symptoms throughout the evaluable interval. Missing days will not be classified as symptom-free days.

Longitudinal analyses will be performed using LMMs, which appropriately account for within-subject correlations arising from repeated measurements, accommodate possible different numbers of observations across participants, also allowing the inclusion of participants with partially missing follow-up data under the Missing At Random (MAR) assumption.

Mixed-effects models are particularly indicated in this case to account for the correlation between repeated observations obtained from the same participant over time. Fixed effects will include study group, time, and their interaction, while participant will be modeled as a random intercept to account for individual baseline differences. Moreover, this approach allows the inclusion of all available observations, accommodates unbalanced follow-up data and missing visits under the MAR assumption, and provides unbiased parameter estimates using maximum likelihood estimation.

The primary model will include as fixed effects:

- study group (epiBD, BD-ADHD, BD-BPD),
- time points (baseline, 6 months, and 12 months),
- group × time interactions.

Clinical and socio-demographic characteristics identified a priori as potential confounders will be included as covariates. The potential influence of medication exposure and other time-varying clinical factors will be examined in prespecified sensitivity analyses, including medication class and dose, treatment changes, adherence, substance use, smoking status, body mass index, sleep quality, medical comorbidities, and current mood severity. Both unadjusted and adjusted estimates will be reported. Given the observational design, residual and time-dependent confounding cannot be excluded, and the resulting associations will not be interpreted as causal.

The significance of the group × time interaction will be used to determine whether longitudinal trajectories differ among the three groups. Estimated marginal means with 95% confidence intervals will be reported, together with standardized effect sizes. The three prespecified pairwise group comparisons will be adjusted using Holm's procedure.

### Secondary analyses

Secondary longitudinal analyses will evaluate changes in different measured clinical features such as mania symptoms, anxiety, impulsivity, quality of life, neuropsychological performance; structural neuroimaging; functional connectivity; inflammatory biomarkers; endocannabinoid markers, tryptophan–kynurenine metabolites; microRNA expression.

Continuous longitudinal outcomes will be analyzed using LMMs, whereas generalized LMMs will be used for non-Gaussian outcomes where appropriate.

Cross-sectional comparisons at each assessment time point will be performed using analysis of covariance (ANCOVA) adjusting for the same predefined covariates.

Neuroimaging and molecular analyses will additionally include relevant technical and biological covariates, such as scanner or recruitment site, intracranial volume, head motion and assay batch. Benjamini–Hochberg false-discovery-rate correction will be applied separately within prespecified families of secondary outcomes. Whole-brain neuroimaging analyses will use imaging-specific familywise-error correction.

### Machine-learning and exploratory analyses

To assess whether multimodal clinical, neuropsychological, neuroimaging, and molecular data can improve the classification of clinical groups and predict longitudinal clinical outcomes, ML analyses will be performed. Specifically, we will consider two main predictive tasks. The first is a multiclass classification task distinguishing epiBD, BD-ADHD, and BD-BPD. The second will involve the prediction of continuous longitudinal outcomes, including burden and mood instability over the 12-months follow-up. Within each training fold, all the variables will undergo standardized preprocessing procedures, including data quality assessment, normalization or standardization of continuous variables, categorical encoding where appropriate, and imputation of missing values using methods appropriate for the data structure. Highly correlated variables and features with near-zero variance will be removed before model fitting. Since the number of features is expected to exceed the number of data points (individuals = 150), dimensionality reduction and feature selection will be applied before model development. Candidate approaches include Least Absolute Shrinkage and Selection Operator (LASSO) and Principal Component Analysis (PCA). Several supervised ML algorithms will be evaluated, including penalized logistic regression (Elastic Net), Random Forests (RF), Support Vector Machines (SVM), and Extreme Gradient Boosting (XGBoost). Model selection will be based on predictive performance while minimizing model complexity to reduce overfitting. Given the relatively limited sample size, predictive performance will be evaluated exclusively through internal validation using repeated nested cross-validation, stratified by group and site where feasible. To prevent information leakage, all data-dependent preprocessing steps, including imputation, scaling, harmonization and batch-effect correction, feature filtering, dimensionality reduction, and feature selection, and all hyperparameter tuning will be performed within the inner cross-validation loop using only the outer training data. The outer test fold will remain completely excluded from model fitting and selection and will be used only for final performance evaluation. When longitudinal observations are included, all data from the same participant will be assigned to the same outer and inner folds. No independent external validation cohort is currently available; therefore, all predictive analyses should be regarded as exploratory. Classification models will be evaluated using different metrics: area under the receiver operating characteristic curve (ROC-AUC), sensitivity, specificity, precision, recall, F1-score, and calibration.

Prognostic performance of the models will be evaluated using root mean squared error (RMSE), mean absolute error (MAE) and the coefficient of determination (*R*^2^).

To improve model interpretability, feature importance will be assessed using permutation importance and Shapley Additive Explanations (SHAP) where computationally appropriate. These analyses will identify the variables contributing most strongly to prediction while facilitating biological interpretation of multimodal signatures.

Because of the high dimensionality of the multimodal datasets relative to the available sample size, all ML analyses are explicitly considered exploratory and hypothesis-generating. Any predictive signatures identified in this study will require replication and external validation in larger independent cohorts before consideration for clinical implementation.

All machine-learning analyses will be performed in Python using established open-source libraries, including scikit-learn, XGBoost, and SHAP.

### Ethical approval

MapCoBD has been approved by the Ethics Committee of Sardinia (protocol 2.10 approved on January 14, 2025) and subsequent approval by the DPO and Health Agency of Cagliari approval #122 on February 17, 2025. This study was conducted in accordance with the Declaration of Helsinki and written informed consent was obtained from all participants prior to enrollment. Recruitment is currently ongoing with 110 individuals tested at T_0_.

## Discussion

MapCoBD is a longitudinal naturalistic study whose primary objective is to compare illness burden, assessed using the prospective NIMH Life Chart Method, and mood instability, derived from daily affective ratings, over 12 months among individuals with epiBD, BD-ADHD, and BD-BPD. Secondary objectives are to characterize cross-sectional and longitudinal differences in clinical, neuropsychological, neuroimaging, and molecular measures across the three prespecified group comparisons. Exploratory multimodal analyses will evaluate whether combinations of these measures can classify clinical group status and predict subsequent illness burden or mood instability. The distinctive contribution of MapCoBD is the direct prospective comparison of three clinically defined BD profiles that have generally been investigated separately. Rather than considering ADHD and BPD comorbidity only as indicators of a more severe or complex form of BD, the study will test whether they are associated with distinguishable longitudinal clinical profiles. Compared with epiBD, both BD-ADHD and BD-BPD are expected to show greater time-integrated illness burden and mood instability. However, the anticipated profiles are not identical: BD-BPD is expected to be characterized by greater affective instability and impulsivity, whereas BD-ADHD is expected to show more prominent attentional and neuropsychological impairment. The direct comparison between BD-ADHD and BD-BPD is particularly relevant because emotional dysregulation, impulsivity, and attentional difficulties may overlap clinically across these conditions, complicating their characterization in individuals with BD. Notably, the longitudinal design will allow us to examine whether changes in multimodal measures precede, accompany, or follow changes in clinical course; however, because the study is observational, these temporal associations will not establish causality. Nevertheless, the main methodological strength of MapCoBD lies in the combination of a prospective longitudinal design with repeated multimodal assessments in three clinically well-characterized BD groups. Daily affective ratings and the NIMH Life Chart Method will provide complementary measures of mood instability and illness burden, while repeated clinical, neuropsychological, neuroimaging, and molecular assessments will allow us to examine whether group differences remain stable or change with the clinical course. The naturalistic design will also capture treatment and illness trajectories under real-world clinical conditions.

By repeatedly assessing the three groups, MapCoBD may help determine whether differences in attention, cognition, impulsivity, and affective instability represent relatively stable features associated with the comorbid profiles or fluctuate according to mood state and overall illness burden. The integration of phenotypic, neuroimaging, and peripheral molecular data will further allow us to explore whether clinically overlapping manifestations in BD-ADHD and BD-BPD are associated with distinct multimodal signatures. In addition, the study will investigate whether baseline or time-varying combinations of clinical and biological features are associated with subsequent mood instability or illness burden. These analyses may generate candidate trajectory models and predictors for subsequent validation in larger, independent samples, but they should be considered exploratory. The potential clinical implications of MapCoBD concern diagnosis, monitoring, and treatment planning. At the diagnostic level, evidence of distinguishable longitudinal profiles could support a more systematic assessment of ADHD and BPD in individuals with BD and improve the clinical differentiation of persistent attentional, impulsive, and emotion-regulation difficulties from symptoms occurring predominantly during affective episodes. This distinction may be particularly important because some manifestations of ADHD, BPD, and BD overlap, while their temporal pattern, persistence, and relationship with mood episodes may differ.

For clinical monitoring, the findings may identify dimensions requiring different emphasis across the three groups. Individuals with BD-BPD may benefit from closer longitudinal monitoring of affective instability, impulsivity, and rapid changes in emotional state, whereas individuals with BD-ADHD may require more systematic assessment of attention, executive functioning, and cognitive difficulties, alongside conventional monitoring of depressive and manic symptoms. Prospective daily ratings and life-chart measures may also help determine whether changes in these dimensions precede periods of increased illness burden and therefore warrant more intensive clinical follow-up. Although MapCoBD is not designed to evaluate treatment efficacy, evidence of distinct clinical trajectories could contribute to more comorbidity-sensitive management strategies. For example, the identification of prominent emotion-regulation and impulsivity-related difficulties in BD-BPD could support greater attention to psychological or rehabilitative interventions targeting these dimensions, whereas persistent attentional and cognitive impairment in BD-ADHD could indicate the need for specific assessment and management of these symptoms, while maintaining appropriate treatment of the underlying BD. In the longer term, validated peripheral, neuroimaging, or multimodal signatures might complement clinical assessment for patient stratification and longitudinal monitoring. However, such signatures should not be considered standalone diagnostic biomarkers or treatment-selection tools without prospective replication, external validation, and evidence of incremental clinical utility beyond standard clinical assessment.

However, some limitations should be acknowledged. The naturalistic observational design does not allow causal inference, and changes in medication, psychotherapy, sleep, substance use, or other clinical factors may influence both illness course and biological measures. In addition, the 12-month follow-up may not capture the full cyclicity of BD, while the intensive assessment schedule may increase missing data and attrition. Finally, the sample size is relatively limited for high-dimensional multimodal and machine-learning analyses; therefore, these analyses will be considered exploratory and any predictive signatures will require external validation.

Collectively, MapCoBD will test whether BD-ADHD and BD-BPD are associated with clinically and biologically distinguishable longitudinal profiles rather than representing only more severe forms of BD. By linking comorbidity-specific clinical trajectories with neuropsychological, neuroimaging, and peripheral molecular measures, the study may provide candidate markers and hypotheses for more precise clinical characterization, monitoring, and comorbidity-informed management, while defining priorities for subsequent confirmatory and intervention studies.
